# Food resource richness increases seed disperser visitations and seed rain richness

**DOI:** 10.1002/ece3.11093

**Published:** 2024-03-04

**Authors:** James P. Holdgrafer, David S. Mason, Tyler Steven Coleman, Marcus A. Lashley

**Affiliations:** ^1^ Department of Wildlife Ecology and Conservation University of Florida Gainesville Florida USA; ^2^ Florida Cooperative Fish and Wildlife Research Unit, Department of Wildlife Ecology and Conservation University of Florida Gainesville Florida USA

**Keywords:** avian seed dispersal, diversity‐maintenance mechanism, mutualism, resource‐tracking, zoochory

## Abstract

Within the context of global change, seed dispersal research often focuses on changes in disperser communities (i.e., seed dispersers, such as birds, in an area) resulting from habitat fragmentation. This approach may not be completely illustrative due to certain seed disperser communities being more robust to fragmentation. Additionally, this top‐down approach overlooks how changing food resources on landscapes impacts resource tracking and, subsequently, seed dispersal. We hypothesized resource tracking may promote diffuse plant–animal dispersal mutualisms if resource richness is positively linked to disperser and seed rain richness. We predicted increasing food resource richness attracts more visits and species of avian dispersers, resulting in higher counts and greater species richness of seeds deposited at sites (i.e., seed rain). We tested this mechanism in two replicated field experiments using a model system with bird feeders positioned above seed traps. In the first experiment, we demonstrated resource presence skews seed rain. In the second experiment, we explored how species richness of food resources (0, 4, 8, or 12 species) affected the species richness and visitation of avian seed dispersers at feeders and in subsequent seed rain. Collectively, we observed a positive relationship between available food resources and seed rain, likely mediated by resource tracking behavior of avian dispersers. Our findings underscore a potential key mechanism that may facilitate ecological diversity, whereby accumulating species richness in the plant community attracts a more diverse seed disperser community and indirectly promotes more species in seed rain. Importantly, the resource tracking mechanism driving this potential positive feedback loop may also result in negative ecosystem effects if global change diminishes resource availability through homogenization processes, such as invasive species colonization. Future research should explore the bottom‐up effects of global change on food resources and seed disperser behavior to complement the literature on changing disperser communities.

## Graphical abstract

Historically, studies on seed network connectivity and health use a top‐down approach, examining the abundance and distribution of seed dispersers on a landscape. In this study, we used a bottom‐up approach to examine how food resource richness at sites impacted the richness of the seed disperser community visiting each site and the richness of the seed community deposited at sites by dispersers.

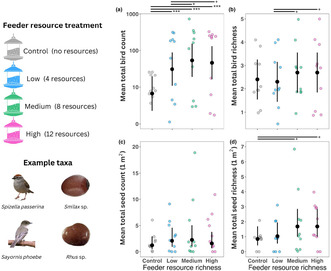

## 1 INTRODUCTION

Although seed dispersal is a key ecological function, we lack a cohesive understanding of how global change affects animal‐mediated seed dispersal in different ecosystems with distinct disperser communities (Teixido et al., 2022). Seed dispersal relationships are generally diffuse mutualisms, defined as a mutually positive relationship including multiple participating species (e.g., pollination and seed dispersal; Gove et al., 2007; Stanton, 2003; Zamora, 2000). In tropical forest biomes, asymmetric declines in the large‐bodied vertebrate participants of diffuse dispersal mutualisms with habitat fragmentation are hypothesized to diminish biodiversity, particularly when some plants are only dispersed by a subset of dispersers (Bovo et al., 2018; Case & Tarwater, 2020; Naniwadekar et al., 2019). More specifically, the loss of large‐bodied frugivores from fragmented landscapes can limit dispersal distances and strengthen dispersal limitation, potentially resulting in negative impacts on larger seeded plants that can only be transported by these large frugivores (Cordeiro & Howe, 2001; Cramer et al., 2007; Jordano et al., 2007). Conversely, seed dispersal by bird communities in temperate region forest biomes may be more robust to habitat fragmentation than in tropical biomes (Bregman et al., 2014; Farwig et al., 2017; but see Fontúrbel et al., 2015). However, much of the seed dispersal research related to anthropogenic factors in temperate forests still focuses on fragmentation (Teixido et al., 2022). Discrepancies like these underscore the need to reevaluate seed dispersal processes for a more robust understanding of how global changes in food resources affect seed dispersal relationships.

Whereas much of the research on fragmentation stresses the top‐down effects of species loss, changes to disperser behavior associated with global changes to food resources may also drive bottom‐up effects on seed dispersal mutualisms. Animal vectors can seek resources that vary in space or time at multiple scales (i.e., resource tracking), which can subsequently influence animal‐mediated seed dispersal (Gleditsch et al., 2017; Mason et al., 2022). Resource‐tracking animals have different diets and may respond to variations in food resources (Blendinger et al., 2015; Fuentes, 1994; Johnson et al., 1985). Moreover, generalist vectors can seek out and subsequently disperse rare fruiting plants (Carlo & Morales, 2016). As a result, resource tracking may result in a positive relationship between the richness of resources and seed rain. Depending on subsequent post‐dispersal interactions and microsite quality associated with such disperser‐preferred resources, this relationship could result in positive feedback that supports diversity in diffuse mutualisms (Gleditsch et al., 2017; Herrera, 1985; Kissling et al., 2007; Mason et al., 2022; Morán‐López et al., 2018; Salazar et al., 2013; Spiegel & Nathan, 2010). Conversely, shifting food resources as a result of climate change, plant invasions, land cover alteration, harvesting, and shifting disturbance regimes (Boyle et al., 2012; Damschen et al., 2019; Fricke & Svenning, 2020; Gleditsch & Carlo, 2011; McConkey & O'Farrill, 2016; McKinney & Lockwood, 1999; Moegenburg & Levey, 2003; Mollot et al., 2017; Rojas et al., 2019) could diminish food resource richness and may influence resource tracking and thus seed dispersal (McConkey & O'Farrill, 2016). Indeed, introduced food resources can disrupt or promote seed dispersal mutualisms (Rojas et al., 2019; Sengupta et al., 2015; Traveset & Richardson, 2006). Therefore, predicting how changing food resources can affect disperser behavior requires understanding how resource richness modulates resource tracking and subsequent patterns of seed dispersal.

We utilized bird feeders as a tool in two experiments to examine how manipulating the richness of food resources influenced (1) the richness and count of birds visiting feeders and (2) the richness and count of seeds deposited beneath feeders. We selected this model system because bird feeders provide a convenient approach to manipulating resource availability, are known to influence bird behavior as resources do, and are ubiquitous in the United States (Cowie & Hinsley, 1988; Fuller et al., 2008; Galbraith et al., 2015; Lepczyk et al., 2004). Rather than measuring avian movement or decision‐making, we use this system to assess whether patterns of avian activity and seed rain are consistent with expectations with fine‐scale resource tracking. In the first experiment, we sought to establish the relationship between resource availability and seed dispersal by birds using our trap design. Previous experiments used similar trap designs or fruiting trees in forests to study seed dispersal (Jordano & Schupp, 2000; Silva et al., 2020). However, we wished to test our specific trap design (bird feeders, mesh‐table traps, and cameras) to establish its efficacy as a sampling method and to establish a baseline dataset to confirm observations in our subsequent experiment were driven by food resource richness, not resource presence. In the second experiment, we measured how differing levels of food resource richness at sites influenced seed disperser and seed rain richness. We expected that increasing the number of resources would lead to more bird counts and greater bird species richness at feeders, generating corresponding patterns in seed rain.

## 2 METHODS

### 2.1 Experiment 1—The effect of food resource presence on seed rain

#### 2.1.1 Location and design

We conducted the initial field experiment in a mixed upland forest in southwest Alabama (32°34′10″ N, 87°57′04″ W) to demonstrate that resources attract seed dispersers and subsequent seed rain. This experiment aided in establishing proof of concept for the efficacy of our seed‐trap design and a baseline to compare the results of Experiment 2 (i.e., The effect of food resource richness on the frequency and richness of seed dispersers and seed rain). Northern cardinals (*Cardinalis cardinalis*) were the most common dispersers on the property and our target plant species was yaupon holly (*Ilex vomitoria*). We randomly placed five pairs of bird feeders (18 × 20 × 23 cm, Ogrmar Hanging Gazebo) approximately 100 m apart in open areas bordering brush. Each pair consisted of one feeder stocked with black oil sunflower seeds (*Helianthus annuus*), while the other feeder remained empty. We placed the feeders on an artificial perch above seed traps that we constructed by affixing 0.5 m^2^ of mesh screening to a frame made from wooden slats. In February 2018, we ran two 14‐day trials. After the first period, we collected seeds from the seed traps and switched the treatments within the pair. Seed count was measured by the total amount of seeds collected within each trap (excluding black oil sunflower seeds) throughout the sampling period.

#### 2.1.2 Statistical analysis

We analyzed seed arrival at traps in R (R Core Team, 2022) with an alpha value of .05 (as were all statistical analyses for this article) using the glmer.nb function to fit a generalized linear model with a negative binomial sampling distribution via maximum likelihood (Bates et al., 2015). We treated resources (baited or empty feeders) and sampling period (1 or 2) as fixed effects and we included pair (1–5) as a random effect to account for within‐pair variation and pseudoreplication (i.e., sacrificial pseudoreplication). The resultant model was formulated as

(1) $${\text{Seeds}}_{i}\sim \mathrm{NB}\left(\mu_{i},\kappa \right)$$

where ${\text{Seeds}}_{i}$ is the negative binomially distributed count of seeds in pairi, $\mu_{i}$ is the expected value for that sample in pairi, and k is the so‐called scaling parameter of the negative binomial distribution (Equation 1). We employ the log‐link function such that the$\;\log_{e}$ (hereafter, “ln”) transformed value of the expected count ($n_{i}$; Equation 2) in pair i is a linear function of the predictors, that is,

(2) $$n_{i}\sim \beta_{0}+\beta_{1}{\text{treat}}_{i}+\beta_{2}{\mathrm{per}}_{i}+b_{i}\sim N\left(\;\mu_{i},\sigma^{2}\right)$$

where $\beta_{0}$ is the intercept value, $\beta_{1,2}$ are the parameter estimates, ${\text{treat}}_{i}$ is the treatment variable for pair i, ${\mathrm{per}}_{i}$ is the sampling period variable for pair i, and $b_{i}\sim N\left(\mu_{i},\sigma^{2}\right)$ is a random‐effects intercept for each pair i that accounts for pair‐specific variation in seed count. We then used the model output to calculate the estimated marginal means and confidence intervals using the emmeans function (Lenth et al., 2022). We also assessed model fit using analysis of variance (Type II) and by calculating the explained variance (e.g., delta, lognormal, and trigamma methods) using the r.squaredGLMM function (Bartoń, 2022). We had one value associated with baited feeders that was an order of magnitude greater than the rest of the upper quantile. We took a conservative approach and removed the pair from that sampling point and conducted the model again.

### 2.2 Experiment 2—The effect of food resource richness on the frequency and richness of seed dispersers and seed rain

#### 2.2.1 Location and design

We conducted the second experiment in North Central Florida on a 9500+ acre biological research station containing a mosaic of carefully managed marshes, oak hardwoods, pine flatwoods, and old‐field habitats in temperate climate conditions (29°40′22″ N, 82°01′58″ W). We established 10 blocks in old fields containing vegetation profiles of primarily grasses and herbaceous plants and two blocks in later successional stages with overstories of pines or successional hardwoods. We spaced blocks approximately 1 km away from one another. During the setup of traps, we recognized signs of wildlife utilizing the old‐field blocks, which included: whitetail deer (*Odocoileus virginianus*), common raccoons (*Procyon lotor*), opossums (*Didelphis virginiana*), squirrels (*Sciurus* spp.), wild turkeys (*Meleagris gallopavo*), mice (*Apodemus* spp.), snakes (Serpentes), and various small perching birds (Passeriformes).

**FIGURE 1:**
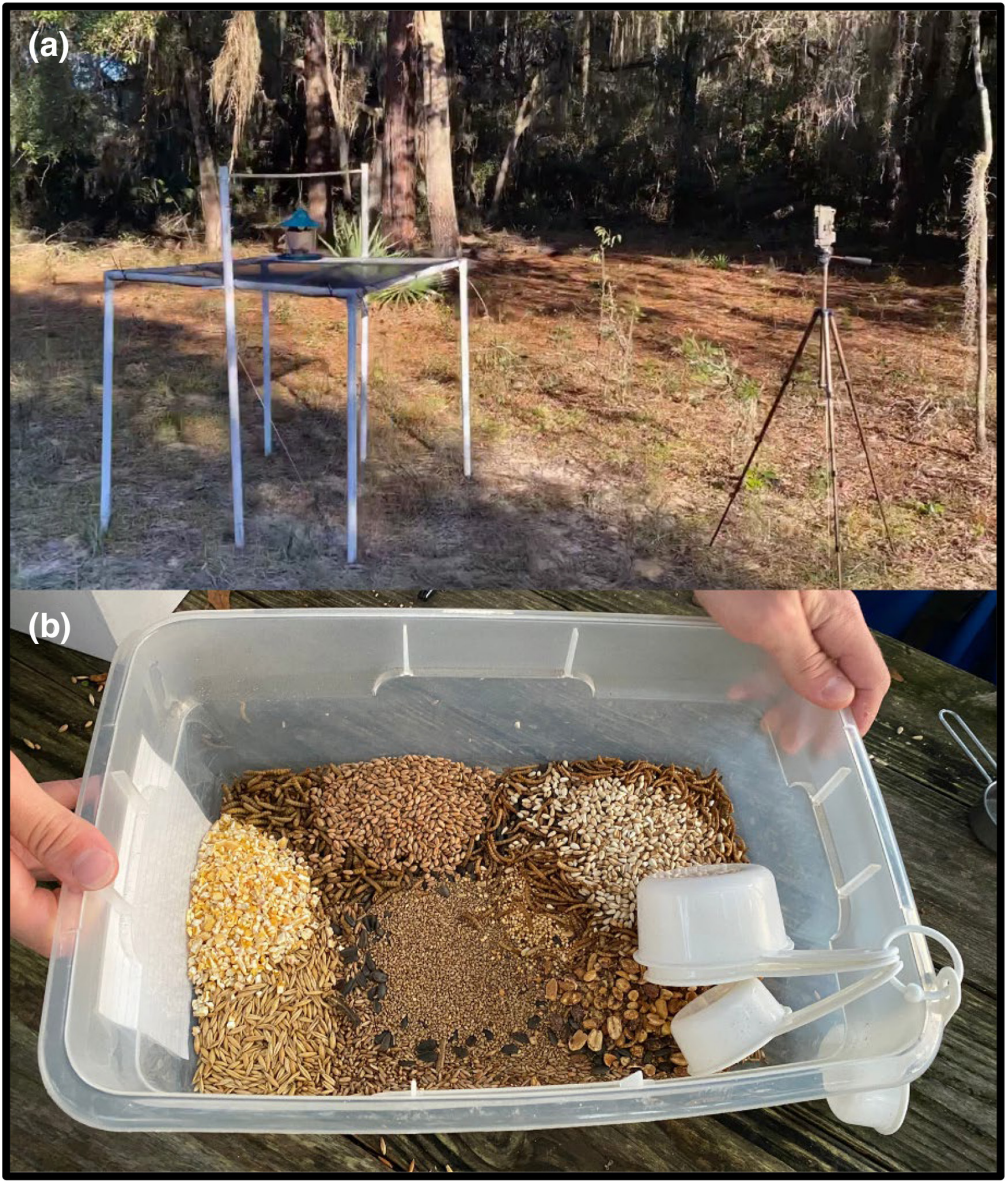
Experiment 2 trap design. (a) We paired seed traps beneath feeders with camera traps to simultaneously monitor seed rain and bird activity. (b) We filled baited feeders with 0.95 L of food resources.

In each block, we placed four seed traps in the corners of a 10 × 10 m square. We constructed the traps from 2.54 cm diameter plastic piping, zip‐ties, pool screen mesh, wooden dowels, and bird feeders. Each trap consisted of a horizontal 1 × 1 m mesh screen zip‐tied to a plastic pipe frame elevated 1 m off the ground by four pipe legs. We suspended each bird feeder above the mesh screen square with a wooden dowel rod horizontally mounted to two vertical 2 m plastic pipes (Figure 1a). The Ogrmar Hanging Gazebo Wild Bird Feeders that we used had dimensions of 18 × 20 × 23 cm and an internal volume of approximately 1.3 L. We labeled each trap with both a trap identification number and its corresponding bird feeder identification number, which was unique to each labeled bird feeder.

Each of our blocks had treatments of zero, four, eight, and 12 food resources within bird feeders. Resources we used in the experiment included: wheat (*Triticum* sp.), rye (*Secale cereale*), brown top millet (*Urochloa ramosa*), white millet (*Panicum miliaceum*), oats (*Avena* sp.), black oil sunflower seeds (*Helianthus annuus*), barley (*Hordeum vulgare*), safflower (*Carthamus tinctorius*), cracked corn (*Zea mays*), Nyjer (*Guizotia abyssinica*), peanuts (*Arachis hypogaea*), and black soldier fly larvae (*Hermetia illucens*). To avoid introducing non‐native species to the research sites, we heat‐treated seeds and nuts to diminish viability and conducted germination tests to examine treatment efficacy. Our heat treatment method consisted of heating seeds with oil contents of 20%–60% at 103°C for 17–24 h and with oil contents below 20% at 130–135°C for 2–4 h. We tested for germination by placing 20 heat‐treated and untreated seeds in damp paper towels within resealable plastic bags stored in dark, room‐temperature conditions for 3 weeks. None of our heat‐treated seeds germinated. We used program R (R Core Team, 2022) to randomly select and assign unique resource combinations for treatments at each block. Then, we used measuring cups to consistently produce a volume of 0.95 L of resources within each bird feeder (Figure 1b), and we stocked control feeders with natural debris and lined the inside with dull‐colored construction paper. At each site, we used a random number generator to assign the placement of treatment levels.

We equipped each seed trap with a Bushnell Trophy Trail Camera (Bushnell Corporation, Overland Park, Kansas, United States) to detect bird activity. We positioned all cameras using tripods 1.14 m from the edge of the seed trap, with the camera lens aligned horizontally and vertically with the bird feeder (approximately 1.50 m above the soil surface; Figure 1a). We placed all cameras facing north or south to minimize non‐bird stimulus from setting off camera traps depending on block‐specific conditions, such as roads or vegetation. We set each trap to high sensitivity with a 10‐min return interval and 15‐s video recording at 720 × 1080‐pixel image resolution.

We sampled all 40 seed traps five times between November 27, 2020 and February 03, 2021. The first three samples occurred weekly, and the final two sampling dates occurred after a 22‐day and then 32‐day interval (i.e., sampling periods are uneven and sample length is confounded with time since the start of the experiment). Our sampling protocol involved visiting each trap, collecting bird scat and loose seeds located anywhere on the trap structure, and placing collections into a labeled resealable plastic bag. In our sampling protocol, “loose seeds” were seeds not stocked within the bird feeders. Then, we counted and attempted to identify the seed samples. Seed count was measured by the total amount of seeds collected within the trap throughout the sampling period, excluding seeds deposited from the stocked bird feeder. We used these collections to create matrices describing the composition of seed communities within the trap at each time point.

Total seed rain was low in the experiment and we did not filter seeds according to the dispersal mechanism. Although many plant species in our data are not typically associated with dispersal by birds, species of plants with no known adaptation for seed dispersal by animals can still be dispersed by birds (Green et al., 2022). Moreover, due to our experimental design, all traps in a block were exposed to the same ambient seed‐rain conditions. We assumed that all traps had an equal chance of receiving seeds from the environment via pathways other than birds and that birds were driving differences in seed rain. Thus, we included all seeds besides those originating from feeders in our final models. To make sure this approach did not produce misleading results, we ran all seed models with the data filtered to exclude species that were less likely dispersed by birds (Appendix S1).

While collecting samples, we also swapped out SD memory cards from our trail cameras monitoring the seed traps. We reviewed the videos and recorded the date, species, and quantity of birds in each video clip. The total bird count for each trap was calculated by adding all the birds observed at a trap over the sampling period. Many of the birds recorded at feeders had mainly granivorous or insectivorous diets. However, birds can disperse seeds via several pathways beyond the consumption of fleshy fruit (e.g., endozoochory), such as dyszoochory (seeds dropped before or during ingestion) or epizoochory (seeds externally transported on animals). Moreover, insectivorous or granivorous birds can still eat fruit and disperse seeds (Whelan et al., 2015). Thus, rather than focusing on frugivorous birds, we included all species in our analyses.

#### 2.2.2 Statistical analysis

We conducted total and time series models for our response variables. We chose to conduct both types of models because resource richness could impact seed dispersal differently depending on the temporal scale. Depending on the response variable, this approach was not always achievable given the data or model performance. A description of our modeling selection and validation process is included in Appendix S2. For all models below, we present estimated marginal means and confidence intervals (Lenth et al., 2022). We also present unadjusted *p*‐values because we did not consider one statistically significant result as a confirmation of our predictions (Rubin, 2021). Such corrections for multiple comparisons could change the inference for some of our results. We thus present comparisons with Holm's correction in Appendix S3.

### 2.3 Bird counts

Before analyzing the data, we filtered out the first 2 weeks of data to allow for an acclimatization period and summed the total bird detections for each trap by week. We then fit a generalized linear model with a negative binomial sampling distribution for counts of total birds at feeders. Our model included feeder resource level treatment (control = 0, low = 4, medium = 8, or high = 12) and weeks since acclimatization (1–10) as categorical fixed effects and block (1–10) as a random effect (Bates et al., 2015). The regression was formulated as

(3) $${\text{Birds}}_{i}\sim \mathrm{NB}\left(\mu_{i},k\right)$$

where ${\text{Birds}}_{i}$ is the negative binomially distributed count of birds detected in block i (Equation 3), $\mu_{i}$ is the expected value for that sample in block i, and k is the negative binomial distribution's scaling parameter. The ln‐transformed value of the expected bird count ($n_{i}$; Equation 4) in block i is a linear function of the predictors, that is,

(4) $$n_{i}∼\beta_{0}+\beta_{1}\text{trea}t_{i}+\beta_{2}{\text{week}}_{i}+b_{i}∼N\;\left(\mu_{i},\sigma^{2}\right)$$

where $\beta_{0}$ is the intercept value, $\beta_{1,2}$ are the parameter estimates, ${\text{treat}}_{i}$ is the treatment variable for block i, ${\text{week}}_{i}$ is the temporal factor variable in block i, and $b_{i}\sim N\left(\mu_{i},\sigma^{2}\right)$ is a random‐effects intercept for each block i that accounts for block‐specific variation in bird count (sacrificial pseudoreplication).

### 2.4 Bird richness

Again, we removed the initial 2 weeks of camera trap data to account for an acclimatization period. We used the lmer function to conduct a linear mixed effects model with a Gaussian sampling distribution to assess total bird species richness at feeders across the experiment (Bates et al., 2015). We treated feeder resource level treatment (control = 0, low = 4, medium = 8, or high = 12) and experiment block (1–10) as fixed and random effects, respectively. The regression was expressed as

(5) $${\text{Rich}}_{i}\sim N\left(\mu_{i},\sigma^{2}\right)$$

where ${\text{Rich}}_{i}$ is the normally distributed range of total bird richness observed in blocki (Equation 5), $\mu_{i}$ is the expected value for that blocki, and $\sigma^{2}$ is the error parameter for the Gaussian distribution. The expected value for total bird richness (Equation 6) in blocki is a linear function of the predictors, that is,

(6) $$n_{i}\sim \beta_{0}+\beta_{1}\text{treat}+b_{i}\sim N\left(\mu_{i},\sigma^{2}\right)$$

where $\beta_{0}$ is the intercept value, $\beta_{1}$ is the parameter estimate, ${\text{treat}}_{i}$ is the treatment variable for blocki, and $b_{i}\sim N\left(\mu_{i},\sigma^{2}\right)$ is a random‐effects intercept for each blocki that accounts for block‐specific variation, and thus pseudoreplication in species richness.

We modeled weekly bird richness as a Poisson process using a maximum likelihood approach with the glmer function (Bates et al., 2015). We treated feeder resource level treatment and block as fixed categorical effects and weeks since acclimatization period (1–10) as a fixed continuous effect. The regression was formulated as

(7) $${\text{Rich}}_{i}\sim \text{Pois}\left(\mu_{i}\right)$$

where ${\text{Rich}}_{i}$ is the Poisson distributed count of weekly bird richness at block i, and $\mu_{i}$ is the expected value and variance for that block i (Equation 7). The ln‐transformed value of weekly bird richness ($n_{i}$ in Equation 8) is a linear function of the predictors, that is,

(8) $$n_{i}∼\beta_{0}+\beta_{1}{\text{treat}}_{i}+\beta_{2}\text{week}X_{i}+\beta_{3}{\text{treat}}_{i}*\text{week}X_{i}∼N\left(\mu_{i},\sigma^{2}\right)$$

where $\beta_{0}$ is the intercept value, $\beta_{1-3}$ are parameter estimates, ${\text{treat}}_{i}$ is the categorical treatment variable at observation i, $\beta_{2}$ is the parameter estimate for the continuous variable of X weeks since acclimatization at observation i, ${\text{treat}}_{i}*\text{week}X_{i}$ is the interaction term, and $b_{i}\sim N\left(\mu_{i},\sigma^{2}\right)$ is a random‐effects intercept for each block i that accounts for block‐specific variation in weekly bird richness (sacrificial pseudoreplication).

### 2.5 Seed counts

We modeled seed counts using the glmer.nb function to fit a generalized linear model with a negative binomial sampling distribution (Bates et al., 2015). We treated resource richness (control = 0, low = 4, medium = 8, or high = 12) and days since start of the experiment (7–75) as fixed categorical and continuous effects, respectively. We included the categorical variable block (1–10) as a random effect to account for within‐block variation and pseudoreplication (i.e., sacrificial pseudoreplication). The resultant model was formulated as

(9) $$\text{Seed}s_{i}\sim \mathrm{NB}\left(\mu_{i},k\right)$$

where $\text{Seed}s_{i}$ is the negative binomially distributed count of seeds counted in block i (Equation 9), $\mu_{i}$ is the expected value for that sample in block i, and k is the scaling parameter of the negative binomial distribution. The ln‐transformed value of the expected seed count ($n_{i}$ in Equation 10) in block i is a linear function of the predictors, that is,

(10) $$n_{i}∼\beta_{0}+\beta_{1}\text{trea}t_{i}+\beta_{2}\text{days}X_{i}+\beta_{3}{\text{treat}}_{i}*\text{days}X_{i}+b_{i}∼N\;\mu_{i},\sigma^{2}$$

where $\beta_{0}$ is the intercept value, $\beta_{1-3}$ are the parameter estimates, $\text{trea}t_{i}$ is the treatment variable in block i, $\text{days}X_{i}$ is the continuous variable in block i for days since start of experiment, ${\text{treat}}_{i}*\text{days}X_{i}$ is the interaction term for block i, and $b_{i}∼N\left(\mu_{i},\sigma^{2}\right)$ is a random‐effects intercept for each block i accounting for block‐specific variation in seed count.

### 2.6 Seed richness

We used the glmer function to conduct a generalized linear mixed effects model with a Poisson sampling distribution to assess total seed species richness at feeders across the experiment (Bates et al., 2015). We treated feeder resource level treatment (control = 0, low = 4, medium = 8, or high = 12) and block (1–10) as fixed and random effects, respectively. The regression was expressed as

(11) $${\text{Rich}}_{i}\sim \text{Pois}\left(\mu_{i}\right)$$

where ${\text{Rich}}_{i}$ is the Poisson distributed value of total seed richness observed in block i, and $\mu_{i}$ is the expected value and error term for that blocki (Equation 11). The expected value for total seed richness ($n_{i}$ in Equation 12) in blocki is a linear function of the predictors, that is,

(12) $$n_{i}\sim \beta_{0}+\beta_{1}\text{treat}+b_{i}\sim N\left(\mu_{i},\sigma^{2}\right)$$

where $\beta_{0}$ is the intercept value, $\beta_{1}$ is the parameter estimate, ${\text{treat}}_{i}$ is the treatment variable for blocki, and $b_{i}\sim N\left(\mu_{i},\sigma^{2}\right)$ is a random‐effects intercept for each blocki that accounts for block‐specific variation, and thus, pseudoreplication in species richness.

We analyzed weekly species richness of seed communities arriving in traps using a generalized linear mixed effects model with a negative binomial sampling distribution via the glmer.nb function (Bates et al., 2015). We treated feeder resource level treatment and block as fixed categorical effects and days since start of the experiment as a fixed continuous effect. This model was formulated as

(13) $$\;{\text{Rich}}_{i}\sim \mathrm{NB}\left(\mu_{i},k\right)$$

where ${\text{Rich}}_{i}$ is the negative binomially distributed count of seed species detected at block i (Equation 13), $\mu_{i}$ is the expected value for that block i, and k is the scaling parameter of the negative binomial distribution. The ln‐transformed value of the expected seed count ($n_{i}$; Equation 14) is a linear function of the predictor variables, that is,

(14) $$n_{i}\sim \beta_{0}+\beta_{1}{\text{treat}}_{i}+\beta_{2}{\mathrm{day}X}_{i}+\beta_{3}{\text{treat}}_{i}*\mathrm{day}X_{i}+b_{i}\sim N\left(\mu_{i},\sigma^{2}\right)$$

where $\beta_{0}$ is the intercept value, $\beta_{1-3}$ are the parameter estimates of the fixed effects, ${\text{treat}}_{i}$ is the fixed treatment variable for block i, ${\mathrm{day}X}_{i}$ is the continuous variable at X days since the experiment started for block i, and ${\text{treat}}_{i}*{\mathrm{day}X}_{i}$ is the feeder resource level treatment and days since start of experiment interaction variable.

## 3 RESULTS

### 3.1 Experiment 1—The effect of food resource presence on seed rain

**FIGURE 2:**
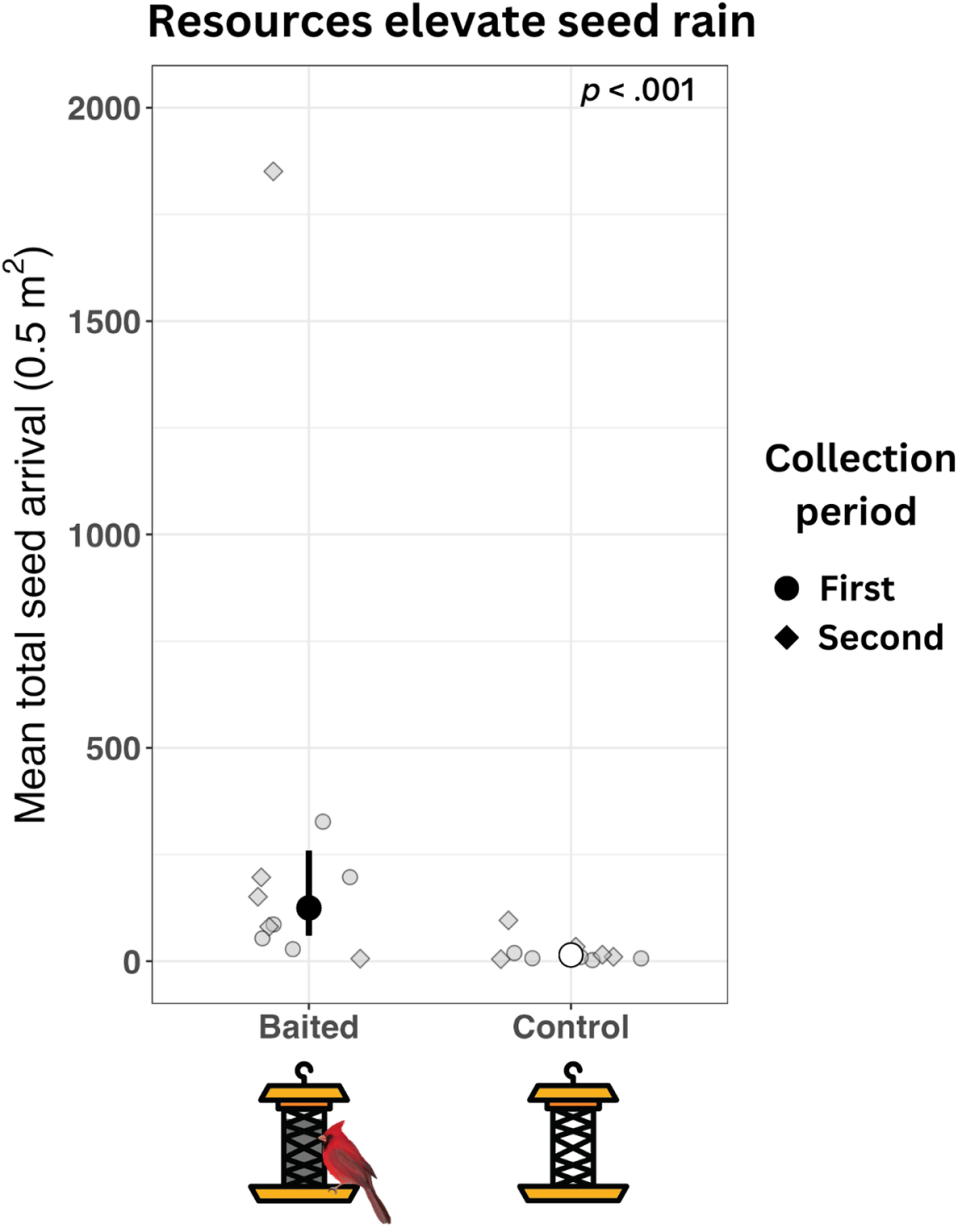
Resources elevate seed rain. In experiment 1, we compared seed rain beneath baited and empty bird feeders to demonstrate that resources attract bird‐dispersed seed rain. Points are estimated marginal means (colored by treatment) and lines are confidence intervals representing the mean number of seeds arriving during each 14‐day sampling event (*y*‐axis). Mean seed arrival beneath baited traps was 11.5 times greater than that beneath control traps.

In the conservative negative binomial model (i.e., dropping a pair with extreme values at the baited feeder), resource presence (*χ* ^2^ = 29.39, *df* = 1, *p* < .001) was a significant predictor of seed arrival but not days since start of experiment (*χ* ^2^ = 2.50, *df* = 1, *p* = .114). The fixed (54%–62%) and random (13%–16%) effects collectively explained 67%–78% of the variance in the conservative seed arrival data. We found that mean seed arrival (Figure 2) in baited traps ($\bar{x}$ = 124.85, LCL = 60.25, UCL = 258.70) was 8.51 times greater (LCL = 3.93, UCL = 18.50, *p* < .001) than control traps ($\bar{x}$ = 14.66, LCL = 6.95, UCL = 30.94). The model without the outlier pair removed produced a similar difference in seed rain with and without resources, but the estimate of mean seed counts for baited feeders was greater ([Link], [Link], [Link]).

### 3.2 Experiment 2—The effect of food resource richness on the frequency and richness of seed dispersers and seed rain

We recorded 4863 bird counts across 10 species on our camera traps. The most counted bird species was the chipping sparrow (*Spizella passerina*, 67.3%), followed by Eastern phoebe (*Sayornis phoebe*, 16.6%), Northern cardinal (*Cardinalis cardinalis*; 14.4%), mourning dove (*Zenaida macroura*; 0.8%), pine warbler (*Setophaga pinus*, 0.6%), gray catbird (*Dumetella carolinensis*, 0.2%), and barred owl (*Strix varia*, 0.1%). Red‐shouldered hawk (*Buteo lineatus*), Eastern screech owl (*Megascops asio*), and sedge wren (*Cistothorus stellaris*) collectively accounted for less than 0.1% of counts.

We distinguished 26 morphotypes from 123 total seeds in our seed traps, with few instances of identification to family, genus, or species. Based on identification and morphology, wind‐dispersed species accounted for two morphotypes (Asteraceae and *Pinus* sp.), and fleshy‐fruited endozoochorous species accounted for three (*Parthenocissus quinquefolia*, *Rhus* sp., and *Smilax* sp.). We also collected one epizoochorous seed associated with mammal dispersal (*Desmodium* sp.) and two canopy species (e.g., *Quercus* and *Pinus* spp.). Collectively, the morphotypes that are not typically associated with seed dispersal by bird species detected in our experiment accounted for 45% of the total seed rain. Many of the remaining unidentified morphotypes were small (<5 mm) hard seeds.

### 3.3 Bird counts

**FIGURE 3:**
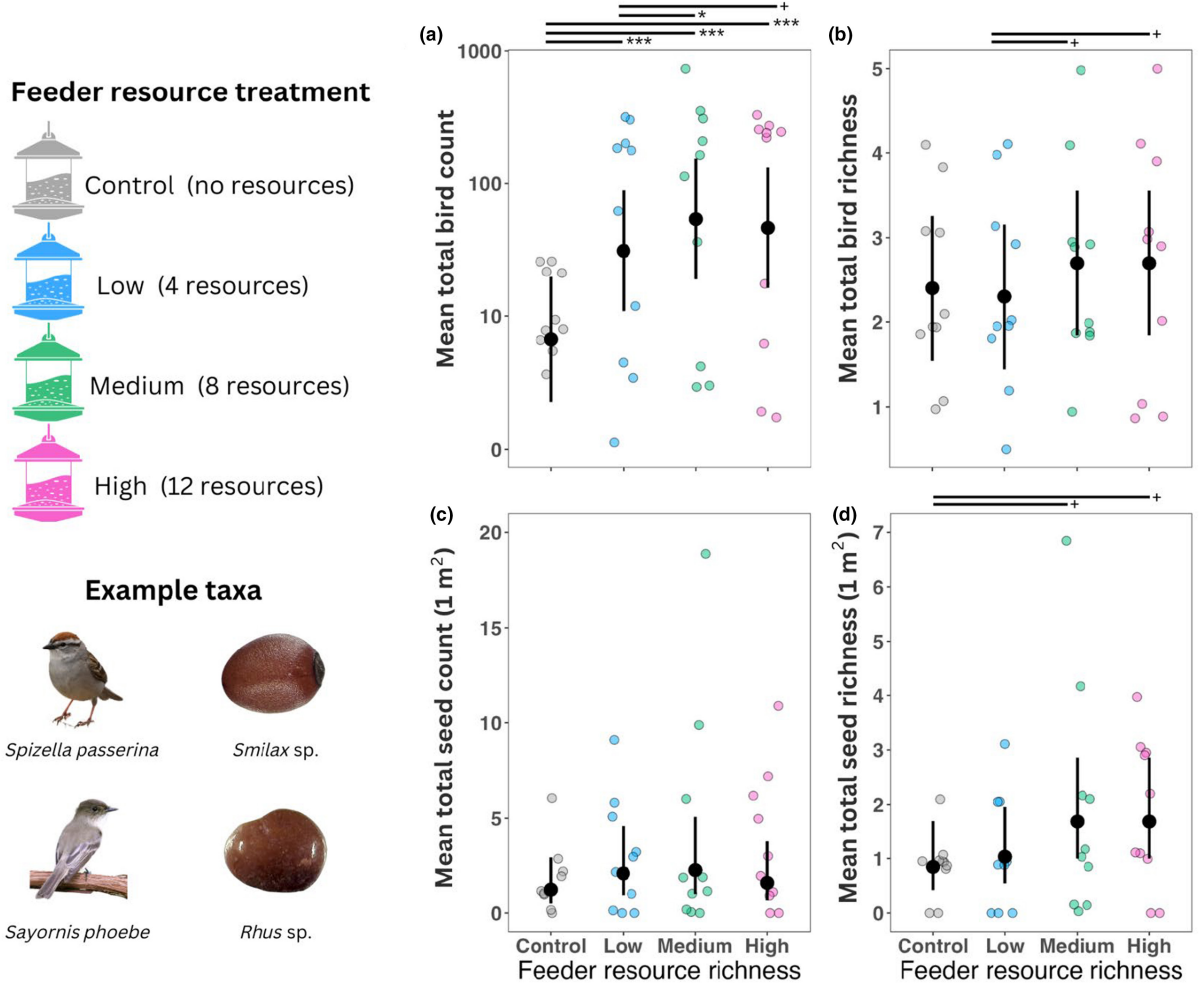
Resource richness drives patterns in bird activity and seed rain. In experiment 2, we measured how resource richness influenced dispersers and seed rain. Black points and lines represent estimated marginal means and confidence limits. Individual data points for each trap are shown with points colored by treatment. For the total seed (68 days) and bird counts (60 days), we multiplied the estimated marginal means and confidence intervals derived from time‐series models by the number of samples. (a) Mean total bird counts at control and low‐resource‐richness feeders were lower than at medium and high‐resource‐richness feeders. Chipping sparrows (*Spizella passerina*) and Eastern phoebes (*Sayornis phoebe*) accounted for 84% of these counts. (b) Mean total bird richness was similar across resource treatments, but weekly bird richness was greater at medium and high‐resource‐richness feeders than control feeders. (c and d) Similarly, mean total seed count and richness were comparable across treatments but increased throughout the experiment more in medium and high‐resource‐richness feeders than control feeders. Of the identified and likely bird‐dispersed seeds collected from our traps, *Rhus* sp. and *Smilax* sp. arrived at traps most often (14% of total seeds counted). Collectively, these results may indicate that resource richness influences spatial patterns of seed dispersal by birds. Symbols indicate significance levels (i.e., *p* < .10 = +, *p* ≤ .05 = *, *p* ≤ .01 = **, and *p* ≤ .001 = ***), and the horizontal lines connect treatments to show pairwise comparisons. Seed images adapted from Tschinkel and Domínguez (2017).

Feeder resource level treatment (*χ* ^2^ = 71.46, *df* = 3, *p* < .001) and weeks since acclimatization period (*χ* ^2^ = 77.73, *df* = 9, *p* < .001) were statistically significant predictors of bird counts at feeders. The random effect of block explained 31%–52% of the variance in bird counts. Treatment and weeks since acclimatization explained 16%–27% (total explained variance = 47%–79%). Weekly bird counts, averaged over all levels of week since acclimatization, were greatest for the medium feeders ($\bar{x}$ = 5.40, LCL = 1.91, UCL = 15.33), followed by high ($\bar{x}$ = 4.64, LCL = 1.63, UCL = 13.18), low ($\bar{x}$ = 3.12, LCL = 1.09, UCL = 8.89), and control ($\bar{x}$ = 0.67, LCL = 0.23, UCL = 1.97; Figure 3a, Table 1).

**TABLE 1:** Total bird counts pairwise comparisons.

Contrast	Ratio	SE	LCL	UCL	*Z* score	*p* Value
Control/low	0.215	0.058	0.127	0.435	−5.748	<.001***
Control/medium	0.124	0.032	0.074	0.247	−7.992	<.001***
Control/high	0.144	0.038	0.086	0.289	−7.335	<.001***
Low/medium	0.577	0.129	0.372	1.041	−2.457	.014*
Low/high	0.671	0.149	0.435	1.204	−1.799	.072^†^
Medium/high	1.164	0.252	0.762	2.060	0.703	.482

*Note*: Symbols indicate significance (i.e., *p* < .10 = ^†^, *p* < .05 = *, *p* < .01 = **, and *p* < .001 = ***).

### 3.4 Bird richness

Treatment was not a significant predictor of total bird species richness at feeders in the overall model (*χ* ^2^ = 5.76, *df* = 3, *p* = .124). The model explained 86% of the variance in total bird species richness. The random effect of block explained most of the variance (84%), with treatment accounting for the remaining 2%. Mean total richness was greatest in the high and medium feeders ($\bar{x}$ = 2.7, UCL = 1.84, LCL = 3.56), followed by control ($\bar{x}$ = 2.4, UCL = 1.54, LCL = 3.26) and low ($\bar{x}$ = 2.3, UCL = 1.44, LCL = 3.16; Figure 3b).

**FIGURE 4:**
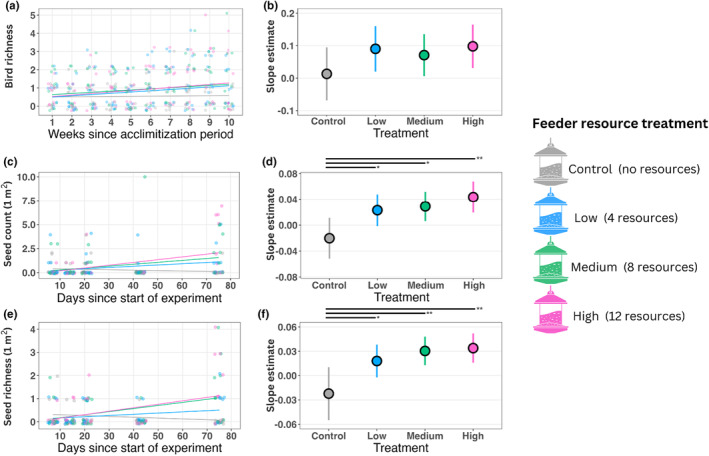
The relationship between resource richness and seed dispersal by birds strengthened as the experiment progressed. In experiment 2, we explored the interaction between resource richness and time (i.e., days since the start of the experiment for seeds and weeks since acclimatization period for birds). The left column shows the linear relationships overlaying raw data, with the line and points colored by treatment. The right column depicts the estimated marginal mean and confidence intervals of the slope of these relationships. (a) Bird richness increased with weeks since acclimatization at feeders with resources, and (b) the interactive effect did not differ among treatments. (c and d) As time progressed, the number of arriving seeds increased for feeders with resources but decreased for the control feeders (e and f) and seed rain richness exhibited a similar pattern. Symbols indicate significance levels (i.e., *p* < .10 = +, *p* ≤ .05 = *, *p* ≤ .01 = **, and *p* ≤ .001 = ***) and are positioned next to horizontal lines that mark pairwise comparisons among treatments.

We found that feeder resource level treatment (*χ* ^2^ = 11.21, *df* = 3, *p* = .011) and weeks since the acclimatization period (*χ* ^2^ = 16.54, *df* = 1, *p* < .001) were statistically significant predictors of weekly bird species richness at feeders. The interaction between treatment and weeks was not significant (*χ* ^2^ = 2.84, *df* = 3, *p* = .418; Figure 4a). The fixed effects in the model explained 4%–6% of the variance in the data. The random blocking effect accounted for 26%–43% of the variance (29%–49% total explained variance). Mean weekly bird richness was greatest at medium feeders ($\bar{x}$ = 0.87, LCL = 0.52, UCL = 1.46), followed by high ($\bar{x}$ = 0.82, LCL = 0.49, UCL = 1.38), low ($\bar{x}$ = 0.75, LCL = 0.44, UCL = 1.26), and control ($\bar{x}$ = 0.54, LCL = 0.32, UCL = 0.93). The coefficient for weeks since acclimatization was greatest for high‐richness feeders (estimate = 0.10, LCL = 0.03, UCL = 0.17), followed by low (estimate = 0.09, LCL = 0.02, UCL = 0.16), medium (estimate = 0.07, LCL = 0.01, UCL = 0.14), and control feeders (estimate = 0.01, LCL = −0.07, UCL = 0.09; Figure 4b). In the weekly bird richness model, simulated residuals deviated from the expected distribution (D = 0.08, *p* = .018). Feeder resource treatment and the treatment and weeks since acclimatization interaction exhibited collinearity (corrected variance inflation factor for treatment = 2.36, weeks since acclimatization = 2.34, interaction term = 2.65, Table 2).

**TABLE 2:** Total and weekly bird richness pairwise comparisons.

Contrast	Total richness						
	Diff	SE	*df*	LCL	UCL	*Z* score	*p* Value
Control/low	0.1	0.21	27	−0.33	0.53	0.475	.638
Control/medium	−0.3	0.21	27	−0.73	0.13	−1.426	.166
Control/high	−0.3	0.21	27	−0.73	0.13	−1.426	.166
Low/medium	−0.4	0.21	27	−0.83	0.03	−1.901	.068^†^
Low/high	−0.4	0.21	27	−0.83	0.03	−1.901	.068^†^
Medium/high	0.0	0.21	27	−0.43	0.43	0.000	1.000

Contrast	Weekly richness						
	Ratio	SE	*df*	LCL	UCL	*Z* score	*p* Value
Control/low	0.727	0.115	∞	0.479	1.105	−2.007	.045*
Control/medium	0.623	0.095	∞	0.416	0.933	−3.090	.002**
Control/high	0.664	0.103	∞	0.440	1.001	−2.629	.009**
Low/medium	0.856	0.121	∞	0.590	1.242	−1.100	.271
Low/high	0.913	0.131	∞	0.625	1.334	−0.634	.526
Medium/high	1.066	0.147	∞	0.741	1.534	0.464	.643

*Note*: Symbols indicate significance (i.e., *p* < .10 = ^†^, *p* < .05 = *, *p* < .01 = **, and *p* < .001 = ***).

### 3.5 Seed counts

Feeder resource level was not a significant predictor of per sample seed counts (*χ* ^2^ = 0.84, *df* = 3, *p* = .839), but days since start of the experiment (*χ* ^2^ = 11.67, *df* = 1, *p* < .001) and the interaction between days since start and feeder resource level were (*χ* ^2^ = 10.01, *df* = 3, *p* = .019; Figure 4c). Collectively, the model explained 6%–41% of the variance in the data. The fixed effects accounted for 3%–22% of this variance, with the remaining 3%–19% attributable to the random effect of block. Mean seed counts were highest for the medium feeders ($\bar{x}$ = 0.45, LCL = 0.20, UCL = 1.01), followed by low ($\bar{x}$ = 0.42, LCL = 0.19, UCL = 0.92), high ($\bar{x}$ = 0.32, LCL = 0.13, UCL = 0.75), and control ($\bar{x}$ = 0.25, LCL = 0.10, UCL = 0.58). The interactive effect of days since start of the experiment was greatest for high feeders (estimate = 0.044, LCL = 0.020, UCL = 0.068), followed by medium (estimate = 0.029, LCL = 0.006, UCL = 0.052), low (estimate = 0.023, LCL = −0.002, UCL = 0.048), and control feeders (estimate = −0.020, LCL = −0.052, UCL = 0.011; Figure 4d). The residuals for this model skewed positively, and the simulated residuals deviated from the expected distribution (D = 0.10, *p* = .034). The model terms exhibited collinearity (corrected variance inflation factor for treatment = 1.70, days since start of experiment = 2.51, and interaction term = 2.08) (Table 3). A model based on filtered data (e.g., no wind‐dispersed, canopy, or epizoochorous seeds) produced similar results ([Link], [Link], [Link]).

**TABLE 3:** Per sample seed counts.

Contrast	Ratio	SE	*df*	LCL	UCL	*Z* score	*p* Value
Control/low	0.590	0.285	∞	0.229	1.520	−1.091	.276
Control/medium	0.544	0.269	∞	0.207	1.430	−1.231	.218
Control/high	0.776	0.403	∞	0.281	2.150	−0.488	.625
Low/medium	0.923	0.428	∞	0.372	2.290	−0.174	.862
Low/high	1.320	0.644	∞	0.504	3.430	0.561	.575
Medium/high	1.439	0.698	∞	0.546	3.720	0.725	.469

Contrast	Interaction with days since start of experiment						
	Diff.	SE	*df*	LCL	UCL	*Z* score	*p* Value
Control/low	−0.043	0.021	∞	−0.084	0.003	−2.084	.037*
Control/medium	−0.049	0.020	∞	−0.088	−0.010	−2.466	.014*
Control/high	−0.064	0.021	∞	−0.104	−0.024	−3.116	.002**
Low/medium	−0.006	0.017	∞	−0.039	0.027	−0.353	.724
Low/high	−0.021	0.017	∞	−0.054	0.013	−1.192	.233
Medium/high	−0.015	0.017	∞	−0.047	0.018	−0.885	.376

*Note*: Symbols indicate significance (i.e., *p* < .10 = ^†^, *p* < .05 = *, *p* < .01 = **, and *p* < .001 = ***).

### 3.6 Seed richness

Treatment was not a significant predictor of total seed richness (*χ* ^2^ = 4.64, *df* = 3, *p* = .201). The entire model explained 18%–29% of the variance in total seed rain richness. The fixed effect of treatment explained 8%–13% of this variance, with the random effect of block accounting for the remaining 10%–17%. Mean total seed richness was greatest at high and medium feeders ($\bar{x}$ = 1.69, LCL = 1.00, UCL = 2.85), followed by low ($\bar{x}$ = 1.03, LCL = 0.55, UCL = 1.96) and control feeders ($\bar{x}$ = 0.85, LCL = 0.42, UCL = 1.70; Figure 3d, Table 4).

**TABLE 4:** Total seed richness.

Contrast	Ratio	SE	*df*	LCL	UCL	*Z* score	*p* Value
Control/low	0.818	0.365	∞	0.341	1.960	−0.450	.6528
Control/medium	0.500	0.203	∞	0.226	1.110	−1.711	.0871^†^
Control/high	0.500	0.203	∞	0.226	1.110	−1.711	.0871^†^
Low/medium	0.611	0.232	∞	0.290	1.290	−1.297	.1948
Low/high	0.611	0.232	∞	0.290	1.290	−1.297	.1948
Medium/high	1.000	0.331	∞	0.523	1.910	0.000	1.0000

*Note*: Symbols indicate significance (i.e., *p* < .10 = ^†^, *p* < .05 = *, *p* < .01 = **, and *p* < .001 = ***).

Resource richness treatment was not a significant predictor of mean seed richness per sample (*χ* ^2^ = 1.44, *df* = 3, *p* = .697), but days since the start of the experiment (*χ* ^2^ = 20.27, *df* = 1, *p* < .001) and the interaction between treatment and days were (*χ* ^2^ = 9.70, *df* = 3, *p* = .021; Figure 4e). The fixed effects in the model explained 4%–23% of the variance in seed richness per sample, with the random effect of block accounting for 1%–7% (5%–30% total). Mean seed per sample richness was greatest in medium feeders ($\bar{x}$ = 0.28, LCL = 0.15, UCL = 0.53), followed by high ($\bar{x}$ = 0.26, LCL = 0.14, UCL = 0.51), low ($\bar{x}$ = 0.23, LCL = 0.12, UCL = 0.45), and control feeders ($\bar{x}$ = 0.18, LCL = 0.09, UCL = 0.38). The interactive effect of days since start of the experiment on mean seed richness per sample was greatest for high feeders (estimate = 0.034, LCL = 0.016, UCL = 0.052), followed by medium (estimate = 0.030, LCL = 0.013, UCL = 0.048), low (estimate = 0.018, LCL = −0.002, UCL = 0.038), and control feeders (estimate = −0.022, LCL = −0.055, UCL = 0.010; Figure 4f). The residuals for the per‐sample richness model were positively skewed, and the predictors exhibited collinearity (corrected variance inflation factor for treatment = 1.95, days since start of the experiment = 1.24, and interaction term = 2.03). Individual observations and blocks influenced the model outcome (Table 5). The model based on the filtered data (e.g., no wind‐dispersed, canopy, or epizoochorous seeds) produced similar results but with more significant differences in total seed richness ([Link], [Link], [Link]).

**TABLE 5:** Per sample seed richness.

Contrast	Ratio	SE	*df*	LCL	UCL	*Z* score	*p* Value
Control/low	0.771	0.358	∞	0.310	1.920	−0.559	.5759
Control/medium	0.643	0.295	∞	0.262	1.580	−0.965	.3346
Control/high	0.687	0.322	∞	0.274	1.720	−0.802	.4228
Low/medium	0.833	0.350	∞	0.366	1.900	−0.435	.6635
Low/high	0.891	0.384	∞	0.383	2.070	−0.269	.7879
Medium/high	1.069	0.454	∞	0.465	2.460	0.157	.8751

Contrast	Interaction with days since start of experiment						
	Diff	SE	*df*	LCL	UCL	*Z* score	*p* Value
Control/low	−0.040	0.020	∞	−0.079	−0.002	−2.053	.040*
Control/medium	−0.053	0.020	∞	−0.090	−0.016	−2.784	.005**
Control/high	−0.056	0.019	∞	−0.094	−0.019	−2.950	.003**
Low/medium	−0.013	0.014	∞	−0.039	0.014	−0.913	.361
Low/high	−0.016	0.014	∞	−0.043	0.011	−1.156	.248
Medium/high	−0.004	0.013	∞	−0.029	0.022	0.273	.785

*Note*: Symbols indicate significance (i.e., *p* < .10 = ^†^, *p* < .05 = *, *p* < .01 = **, and *p* < .001 = ***).

## 4 DISCUSSION

Our experiments provide some support for our prediction that a positive relationship between food‐resource and seed‐rain species richness is mediated by resource‐tracking avian dispersers. However, the inconsistency and strength of these relationships suggest that factors beyond resources also influence seed rain. Moreover, our samples included few seeds and birds, many of which were not typically associated with animal‐mediated dispersal. Still, whereas previous research has been grounded in the top‐down effect of losing species (e.g., see Cramer et al., 2007; Donoso et al., 2020; Naniwadekar et al., 2019; Pérez‐Méndez et al., 2016), we present a bottom‐up framework based on the food resources that support diversity in diffuse mutualisms. This framework theorizes that naturogenic or anthropogenic landscape changes that affect the abundance and richness of food resources may have an impact on seed dispersal mutualisms.

We demonstrated increased seed‐dispersing bird activity with resource availability, which is consistent with previous studies on resource tracking (Gleditsch et al., 2017). Although overall bird richness was low in our second experiment, we also documented a positive response in weekly disperser visits and species richness at feeders with increasing resource options. Previous research indicates that animals participating in diffuse seed dispersal relationships seek diverse resources to maintain healthy nutritional levels, which may incentivize seed dispersers to seek rare resources (Amato et al., 2013; Blendinger et al., 2022; Carlo & Morales, 2016). Thus, our results may indicate that increased food resource richness can be effective for recruiting dispersers to sites even without many disperser species in the community. The observed patterns in bird visitations and richness may also be explained by competitive interactions among species or individuals at feeders that stabilize total species richness even as visitations increase (Galbraith et al., 2017; Miller et al., 2017). Collectively, our observations of bird dispersers at feeders inform one aspect of a complex relationship among resource richness, disperser diet, and disperser interactions that drive the activity of bird communities at food resources.

We observed a positive relationship between the presence of resources and seed rain, which echoes previous work demonstrating the indirect effects of resources and attractive wildlife locations on plant communities (Archibald et al., 2005; Boggess et al., 2021; Carlo & Morales, 2016; Gleditsch et al., 2017; Kwit et al., 2004; Mason et al., 2022; Rodríguez‐Pérez et al., 2014; Salazar et al., 2013). We speculate that resources can gradually and indirectly influence plant communities over time by increasing the amount and species richness of seed rain. Additionally, the positive relationship between total seed rain and resource richness increased even as seed counts plateaued at medium resource feeders, which may indicate that high resource richness (e.g., 12 resource species) attract individual dispersers with intraspecific traits that encourage the dispersal of more plant species (Zwolak, 2018). Importantly, our resource treatments incorporate a variety of sources (e.g., fruit, seeds, and insects), which more accurately reflects the broad diet of potential avian dispersers in our system. The resultant pattern of resource tracking on seed rain thus confirms the expectation that cross‐resource type interactions may influence seed dispersal (Gleditsch et al., 2017). As such, resources resulting from community diversity more generally (i.e., beyond fruit‐producing plants) may also influence seed rain, with potential downstream consequences for plant communities and the interactions they support.

The positive relationship we documented among food‐resource and seed‐rain species richness may indicate that the arrangement and richness of resources influence seed dispersal. If resulting in colonization, increased seed rain richness toward resource‐rich locations may translate into greater resource and disperser species richness, promoting diversity in a diffuse mutualism (Carlo & Morales, 2016; Kissling et al., 2007; Morán‐López et al., 2018). However, primary food resources for dispersers (e.g., plant tissues and insects) are changing worldwide with land use conversion, climate change, introduced species, harvesting, and altered disturbance regimes (Bowler et al., 2019; Boyle et al., 2012; Damschen et al., 2019; Gleditsch & Carlo, 2011; McConkey & O'Farrill, 2016; Moegenburg & Levey, 2003; Mollot et al., 2017; Sengupta et al., 2015). For example, invasive species may homogenize local food resources, disrupting resource tracking in animals or reducing the potential species richness of seed rain (Fricke & Svenning, 2020; McKinney & Lockwood, 1999). Over long timescales, such declines in seed rain diversity could have substantial effects on vegetation communities, especially once native seed banks are depleted (Plue et al., 2021). Thus, our findings suggest that understanding the effects of global change on seed dispersal requires examining resource availability and species richness to supplement the conventional focus on habitat fragmentation and disperser communities.

Limitations and weak statistical results in our work add ambiguity to our conclusions. The small differences in seed rain across resource‐richness treatments that we documented could have resulted from contamination in the feeder resources or from mammals (e.g., raccoons) visiting the feeders, both of which would indicate that we did not demonstrate linkage between resource richness and bird‐mediated seed dispersal. Moreover, our attempts to assess this linkage are complicated by the identity of the birds and seeds arriving at our traps. Granivorous and insectivorous species dominated most bird activity. Similarly, we classified relatively few arriving seeds as belonging to fleshy‐fruited plant species. Still, many overlooked dispersers and plants participate in diffuse dispersal mutualisms beyond the frugivorous birds and fleshy‐fruited species typically associated with dispersal (Green et al., 2022; Whelan et al., 2015). Even if the relationship between resources and bird‐mediated seed dispersal that we demonstrate is indicative of natural processes, the amount of seed rain we observed is unlikely to have a biologically significant impact on communities at the spatial or temporal scales simulated by our experiment. On the other hand, the effect of resource richness on this dispersal mutualism may be stronger than our data indicate but masked by our sampling methodology. We collected data during the dormant season, which likely affected the availability of avian dispersers and seeds. Given the positive interactive trends in seed rain among resource richness levels and the time since the start of the experiment, we might expect a stronger positive relationship among resources and seed dispersal with longer experiments or greater exposure to seed or disperser diversity. However, our evidence does not address such a scenario. Instead, we provide evidence that should encourage future research exploring the effect of resource diversity on seed dispersal, but we caution against overextrapolation from our results. Finally, we note that we focused on resource tracking at fine scales. Variations in temporal and spatial scales may influence relationships among resource presence and richness and seed dispersal.

Although evidence of resource tracking by seed dispersers is widespread, dispersal research related to global change has often focused on the loss of dispersers rather than changes in disperser behavior (Gleditsch et al., 2017; McConkey & O'Farrill, 2016; Valiente‐Banuet et al., 2015; but see Gleditsch & Carlo, 2011; Moegenburg & Levey, 2003; Rojas et al., 2019; Sengupta et al., 2015). Our work illustrates that frameworks primarily built upon the top‐down effects of losing large‐bodied vertebrate dispersers may overlook the integral bottom‐up role that food resources play in shaping seed dispersal patterns (Bregman et al., 2014; McConkey & O'Farrill, 2016). Instead, assessing changes to food resources may provide integral information for efforts to conserve and manage seed dispersal networks. Future research should thus focus on addressing how the role of food resource quality and composition impacts seed dispersal networks, especially in situations where disperser food resources are threatened by global change.

## AUTHOR CONTRIBUTIONS

**James P. Holdgrafer:** Conceptualization (equal); data curation (equal); investigation (equal); methodology (equal); project administration (equal); resources (equal); supervision (equal); validation (equal); writing – original draft (lead); writing – review and editing (lead). **David S. Mason:** Conceptualization (equal); data curation (equal); formal analysis (lead); investigation (equal); methodology (equal); project administration (equal); supervision (equal); validation (equal); writing – original draft (equal); writing – review and editing (equal). **Tyler Steven Coleman:** Formal analysis (lead); software (equal); validation (equal); writing – review and editing (supporting). **Marcus A. Lashley:** Conceptualization (equal); funding acquisition (lead); methodology (equal); project administration (equal); resources (lead); supervision (supporting); writing – review and editing (supporting).

## CONFLICT OF INTEREST STATEMENT

The authors have no conflict of interest to declare.

## Supporting information


Appendix S1.

Appendix S2.

Appendix S3.

## Data Availability

The data and code used in this article are openly available on GitHub: https://github.com/tscoleman3/fl_bird_feeder.
