# Supplementary material for: Food resource richness increases seed disperser visitations and seed rain richness
Source: Ecol Evol. 2024 Mar 4;14(3):e11093. doi: 10.1002/ece3.11093 (PMC10911962; doi:10.1002/ece3.11093)
Supplement: Supplementary file 1 — Appendix S1. [file ECE3-14-e11093-s003.docx]

**Supplementary materials. Alternate models.**

Experiment 1

We ran a negative binomial model using the glmer.nb function with the extreme outlier, again with treatment (baited and empty) and sampling period (1 or 2) as categorical variables and pair (1–5) as a random blocking effect (Bates et al., 2015).

Resource presence (χ2 = 41.19, df = 1, p < 0.001) and sampling period (χ2 = 7.52, df = 1, p < 0.006) were again statistically significant predictors of seed arrival (i.e., count), collectively accounting for 57-64% of model variance in the full model. The random effect of pair explained 17-19% of the remaining variance in seed arrival (74-83% total). Mean seed arrival in baited traps (x̄ = 161.58; LCL = 70.74, UCL = 369.05) was 11.53 times greater (LCL = 5.47, UCL = 24.30, p < 0.001) than control traps (x̄ = 14.01, LCL = 6.07, UCL = 32.33).

Experiment 2

We ran models with the seed rain data filtered to exclude morphotypes with adaptations for wind-dispersal (Asteraceae), canopy tree species (i.e., *Quercus* and *Pinus* spp.), and epizoochorous species adapted for dispersal by mammals (i.e., *Desmodium* sp.). The structure of these models was identical to the respective models for each analysis in the manuscript. Below, we briefly discuss similarities and differences with the models on unfiltered data. Then, we present the output from the emmeans, emtrends, and confint functions (Lenth, 2022).

**2.3 Seed counts**

We ran the seed count model using the glmer.nb function to fit a generalized linear model with a negative binomial model (Bates et al., 2015). Resource richness treatment (control = 0, low = 4, medium = 8, or high = 12) was a categorical fixed effect, days since start of the experiment (7–75) was a continuous fixed effect, and block (1–10) was a random effect.

The full model did not converge, but the analysis of variance produced results consistent with the unfiltered data (feeder treatment: χ2 = 0.61, df = 3, p = 0.894; days since start of experiment: χ2 = 20.02, df = 1 p < 0.001; interaction term: χ2 = 7.89, df = 3, p = 0.048). In the unfiltered data, none of the pairwise contrasts were significant. With the filtered data, the pairwise contrast between control and medium feeders was marginally significant without Holm’s correction for multiple comparisons (ratio = 0.24, LCL = 0.05, UCL = 1.14, p = 0.073).

| **Treatment** | **Mean** | **SE** | **LCL** | **UCL** |
| --- | --- | --- | --- | --- |
| Control | 0.03 | 0.03 | 0.00 | 0.17 |
| Low | 0.09 | 0.06 | 0.02 | 0.37 |
| Medium | 0.12 | 0.08 | 0.03 | 0.47 |
| High | 0.02 | 0.03 | 0.00 | 0.22 |

| **Contrast** | **Effect size** | **SE** | **LCL** | **UCL** | **p value** |
| --- | --- | --- | --- | --- | --- |
| Control/High | 1.15 | 1.37 | 0.05 | 26.56 | 1.000 |
| Control/Low | 0.32 | 0.27 | 0.03 | 2.30 | 0.723 |
| Control/Medium | 0.24 | 0.19 | 0.03 | 1.96 | 0.437 |
| High/Low | 0.28 | 0.30 | 0.02 | 4.76 | 0.723 |
| High/Medium | 0.21 | 0.22 | 0.01 | 3.24 | 0.655 |
| Low/Medium | 0.74 | 0.46 | 0.14 | 3.81 | 1.000 |

Mean seed counts and pairwise comparisons with Holm’s correction

| **Contrast** | **Ratio** | **SE** | **LCL** | **UCL** | **p value** |
| --- | --- | --- | --- | --- | --- |
| Control/High | 1.15 | 1.37 | 0.11 | 11.85 | 0.905 |
| Control/Low | 0.32 | 0.27 | 0.06 | 1.69 | 0.181 |
| Control/Medium | 0.24 | 0.19 | 0.05 | 1.14 | 0.073 |
| High/Low | 0.28 | 0.30 | 0.03 | 2.30 | 0.236 |
| High/Medium | 0.21 | 0.22 | 0.03 | 1.60 | 0.131 |
| Low/Medium | 0.74 | 0.46 | 0.22 | 2.50 | 0.629 |

Means seed counts and pairwise comparisons without Holm’s correction

Pairwise contrasts were comparable to the filtered data. With correction, the only significant contrast was between control and high feeders (estimate = -0.119, LCL = -0.233, UCL = -0.005, p = 0.035), and without, between control and medium (estimate = -0.075, LCL = -0.148, UCL = - 0.001, p = 0.046) and control and low (estimate = -0.086, LCL = -0.162, UCL = -0.010, p = 0.026).

| **Treatment** | **Trend** | **SE** | **LCL** | **UCL** |
| --- | --- | --- | --- | --- |
| Control | -0.035 | 0.035 | -0.103 | 0.033 |
| Low | 0.051 | 0.017 | 0.018 | 0.084 |
| Medium | 0.040 | 0.014 | 0.013 | 0.066 |
| High | 0.084 | 0.005 | 0.034 | 0.133 |

| **Contrast** | **Difference** | **SE** | **LCL** | **UCL** | **p value** |
| --- | --- | --- | --- | --- | --- |
| Control/High | -0.119 | 0.043 | -0.233 | -0.005 | 0.035 |
| Control/Low | -0.086 | 0.039 | -0.188 | 0.016 | 0.130 |
| Control/Medium | -0.075 | 0.038 | -0.174 | 0.024 | 0.186 |
| High/Low | 0.033 | 0.030 | -0.047 | 0.112 | 0.555 |
| High/Medium | 0.044 | 0.028 | -0.031 | 0.119 | 0.362 |
| Low/Medium | 0.011 | 0.021 | -0.044 | 0.067 | 0.586 |

Interaction and pairwise comparisons with Holm’s correction

| **Contrast** | **Difference** | **SE** | **LCL** | **UCL** | **p value** |
| --- | --- | --- | --- | --- | --- |
| Control/High | -0.119 | 0.043 | -0.203 | -0.034 | 0.006 |
| Control/Low | -0.086 | 0.039 | -0.162 | -0.010 | 0.026 |
| Control/Medium | -0.075 | 0.038 | -0.148 | -0.001 | 0.046 |
| High/Low | 0.033 | 0.030 | -0.026 | 0.091 | 0.278 |
| High/Medium | 0.044 | 0.028 | -0.012 | 0.100 | 0.121 |
| Low/Medium | 0.011 | 0.021 | -0.030 | 0.053 | 0.586 |

Interaction and pairwise comparisons without Holm’s correction

**2.4 Seed richness**

We used the glmer function to conduct a general linear mixed effects model with a Poisson sampling distribution (Bates et al., 2015). We again treated feeder resource level treatment (control = 0, low = 4, medium = 8, or high = 12) and block (1–10) as fixed and random effects, respectively.

Treatment was again not a statistically significant predictor of mean total species richness of plants arriving at the traps in the full model (χ2 = 6.05, df = 3, p = 0.109). Without correcting for multiple comparisons, the contrast between control and medium (estimate = 0.29, LCL = 0.09, UCL = 0.87, p = 0.027) and control and high (estimate = 0.31, LCL = 0.10, UCL = 0.94, p = 0.039) was significant. These contrasts were marginally significant using the unfiltered data.

| **Treatment** | **Mean** | **SE** | **LCL** | **UCL** |
| --- | --- | --- | --- | --- |
| Control | 0.25 | 0.17 | 0.07 | 0.91 |
| Low | 0.51 | 0.28 | 0.17 | 1.48 |
| Medium | 0.89 | 0.44 | 0.34 | 2.35 |
| High | 0.83 | 0.41 | 0.31 | 2.20 |

| **Contrast** | **Ratio** | **SE** | **LCL** | **UCL** | **p value** |
| --- | --- | --- | --- | --- | --- |
| Control/High | 0.31 | 0.18 | 0.07 | 1.39 | 0.196 |
| Control/Low | 0.50 | 0.31 | 0.10 | 2.52 | 0.827 |
| Control/Medium | 0.29 | 0.16 | 0.06 | 1.28 | 0.163 |
| High/Low | 1.62 | 0.73 | 0.50 | 5.32 | 0.827 |
| High/Medium | 0.93 | 0.36 | 0.34 | 2.57 | 0.847 |
| Low/Medium | 0.57 | 0.25 | 0.18 | 1.84 | 0.827 |

Means total richness and pairwise comparisons with Holm’s correction

| **Contrast** | **Ratio** | **SE** | **LCL** | **UCL** | **p value** |
| --- | --- | --- | --- | --- | --- |
| Control/High | 0.31 | 0.18 | 0.10 | 0.94 | 0.039 |
| Control/Low | 0.50 | 0.31 | 0.15 | 1.66 | 0.258 |
| Control/Medium | 0.29 | 0.16 | 0.09 | 0.87 | 0.027 |
| High/Low | 1.62 | 0.73 | 0.67 | 3.92 | 0.280 |
| High/Medium | 0.93 | 0.36 | 0.44 | 1.98 | 0.847 |
| Low/Medium | 0.57 | 0.25 | 0.24 | 1.36 | 0.207 |

Means total richness and pairwise comparisons without Holm’s correction

We used the glm.nb function to run a generalized linear mixed effects model with a negative binomial sampling distribution to examine the species richness of seed communities arriving in traps per sampling period (Bates et al., 2015). We again treated feeder resource level treatment and block as fixed and random categorical effects, respectively. Days since start of the experiment (7–75) was a continuous fixed effect.

Consistent with the unfiltered data, treatment was not a significant predictor (χ2 = 2.29, df = 3, p = 0.515) in the full model, but sampling period (χ2 = 23.21, df = 1, p < 0.001) and the interaction term were (χ2 = 8.70, df = 3, p = 0.034). Again, none of the pairwise comparisons of mean seed detections were significant with or without correction for multiple comparisons. This model had convergence issues.

| **Treatment** | **Mean** | **SE** | **LCL** | **UCL** |
| --- | --- | --- | --- | --- |
| Control | 0.04 | 0.03 | 0.01 | 0.18 |
| Low | 0.08 | 0.05 | 0.02 | 0.25 |
| Medium | 0.10 | 0.06 | 0.03 | 0.31 |
| High | 0.03 | 0.03 | 0.00 | 0.19 |

| **Contrast** | **Ratio** | **SE** | **LCL** | **UCL** | **p value** |
| --- | --- | --- | --- | --- | --- |
| Control/High | 1.48 | 1.62 | 0.08 | 26.44 | 1.000 |
| Control/Low | 0.54 | 0.41 | 0.07 | 4.08 | 1.000 |
| Control/Medium | 0.42 | 0.32 | 0.06 | 3.14 | 1.000 |
| High/Low | 0.36 | 0.36 | 0.03 | 5.04 | 1.000 |
| High/Medium | 0.29 | 0.28 | 0.02 | 3.90 | 1.000 |
| Low/Medium | 0.79 | 0.49 | 0.16 | 4.00 | 1.000 |

Mean seed richness and pairwise comparisons with Holm’s correction

| **Contrast** | **Ratio** | **SE** | **LCL** | **UCL** | **p value** |
| --- | --- | --- | --- | --- | --- |
| Control/High | 1.48 | 1.62 | 0.17 | 12.60 | 0.719 |
| Control/Low | 0.54 | 0.41 | 0.12 | 2.42 | 0.418 |
| Control/Medium | 0.42 | 0.32 | 0.10 | 1.87 | 0.257 |
| High/Low | 0.36 | 0.36 | 0.05 | 2.56 | 0.309 |
| High/Medium | 0.29 | 0.28 | 0.04 | 1.99 | 0.206 |
| Low/Medium | 0.79 | 0.49 | 0.24 | 2.64 | 0.700 |

Mean seed richness and pairwise comparisons without Holm’s correction

Similar to the unfiltered model, the pairwise comparison of the days since start of experiment and resource richness interaction were again significant for control and high resource richness feeders (estimate = -0.105, LCL = -0.204, UCL = -0.006, p = 0.030). Contrary to the unfiltered model, the pairwise comparison between control and medium was only significant without correcting for pairwise comparisons (estimate = -0.073, LCL = -0.137, UCL = -0.010, p = 0.024). Moreover, without correction, the contrast between control and low that was significant in the unfiltered model was only marginally significant (estimate = -0.058, LCL = -0.123, UCL = 0.008, p = 0.084). However, the contrast between high and low was marginally significant but it was not with the unfiltered data (estimate = 0.048, LCL = -0.003, UCL = 0.098, p = 0.064).

| **Treatment** | **Trend** | **SE** | **LCL** | **UCL** |
| --- | --- | --- | --- | --- |
| Control | -0.029 | 0.030 | -0.089 | 0.031 |
| Low | 0.028 | 0.014 | 0.002 | 0.055 |
| Medium | 0.044 | 0.012 | 0.021 | 0.067 |
| High | 0.076 | 0.022 | 0.033 | 0.119 |

| **Contrast** | **Difference** | **SE** | **LCL** | **UCL** | **p value** |
| --- | --- | --- | --- | --- | --- |
| Control/High | -0.105 | 0.037 | -0.204 | -0.006 | 0.030 |
| Control/Low | -0.058 | 0.033 | -0.145 | 0.030 | 0.256 |
| Control/Medium | -0.073 | 0.033 | -0.159 | 0.013 | 0.121 |
| High/Low | 0.048 | 0.026 | -0.020 | 0.116 | 0.256 |
| High/Medium | 0.032 | 0.025 | -0.034 | 0.097 | 0.398 |
| Low/Medium | -0.016 | 0.018 | -0.063 | 0.031 | 0.398 |

Interaction and pairwise comparisons with Holm’s correction

| **Contrast** | **Difference** | **SE** | **LCL** | **UCL** | **p value** |
| --- | --- | --- | --- | --- | --- |
| Control/High | -0.105 | 0.037 | -0.178 | -0.032 | 0.005 |
| Control/Low | -0.057 | 0.033 | -0.123 | 0.008 | 0.084 |
| Control/Medium | -0.073 | 0.033 | -0.137 | -0.010 | 0.024 |
| High/Low | 0.048 | 0.026 | -0.003 | 0.098 | 0.064 |
| High/Medium | 0.032 | 0.025 | -0.017 | 0.080 | 0.199 |
| Low/Medium | -0.016 | 0.018 | -0.051 | 0.019 | 0.377 |

Interaction and pairwise comparisons without Holm’s correction
