# Supplementary material for: Food resource richness increases seed disperser visitations and seed rain richness
Source: Ecol Evol. 2024 Mar 4;14(3):e11093. doi: 10.1002/ece3.11093 (PMC10911962; doi:10.1002/ece3.11093)
Supplement: Supplementary file 2 — Appendix S2. [file ECE3-14-e11093-s001.docx]

**Model selection approach**

We determined differences in mean counts and richness for birds and seed rain, and we evaluated potential linear relationships in these response variables as the experiment progressed. We developed a standardized model selection process using general linear models via maximum likelihood:

1. Construct a full model including a random effect for block and an interaction term with a continuous temporal variable.
2. If those models did not converge or performed poorly, replace the random effect for block with a fixed effect.
3. If these models did not converge or performed poorly, drop the interaction term, and construct a model using a categorical temporal variable and a random effect for block.
4. If models perform similarly, choose the model that incorporates the blocked experimental design and interaction term.

We determined the distribution of the data using the descdist function (Delignette-Muller & Dutang, 2015) and we explored model quality with the check_model function (Lüdecke et al., 2021), the influence function (Nieuwenhuis et al., 2012), and with simulated residuals using the Dharma package (Hartig, 2022). We assessed selected models using type II analysis of variance and by calculating explained variance. We calculated explained variance using the r.squaredGLMM function (e.g., delta, lognormal, and trigamma methods) for mixed effects models (Bartoń, 2022) and McFadden's pseudo-R2 using the pR2 function for models without random effects (Jackman, 2020). Then, we calculated and compared estimated marginal means and trends with confidence intervals and pairwise comparisons using the emmeans, emtrends, and confint functions (Lenth et al., 2022). We reported significant pairwise contrasts derived with and without Holm’s correction for multiple comparisons. If contrasts were significant, we also reported all effect sizes. We reported effect sizes based on Holm’s correction unless the pairwise comparisons were only significant without correction. If models showed collinearity, we reported generalized variance inflation factors calculated using the vif function (Fox & Weisberg, 2019).
