# Supplementary material for: Food resource richness increases seed disperser visitations and seed rain richness
Source: Ecol Evol. 2024 Mar 4;14(3):e11093. doi: 10.1002/ece3.11093 (PMC10911962; doi:10.1002/ece3.11093)
Supplement: Supplementary file 3 — Appendix S3. [file ECE3-14-e11093-s002.docx]

**Supplementary materials. Holm’s corrections for pairwise comparisons**

| **Total bird counts** | | | | | | | | |
| --- | --- | --- | --- | --- | --- | --- | --- | --- |
|  |  | | | | | | |  |
| **Contrast** | **Ratio** | **SE** | ***df*** | **LCL** | **UCL** | **Z score** | **P value** |  |
| Control / Low | 0.215 | 0.058 | ∞ | 0.127 | 0.435 | -5.748 | <0.001 | ^***^ |
| Control / Medium | 0.124 | 0.032 | ∞ | 0.074 | 0.247 | -7.992 | <0.001 | ^***^ |
| Control / High | 0.144 | 0.038 | ∞ | 0.086 | 0.289 | -7.335 | <0.001 | ^***^ |
| Low / Medium | 0.577 | 0.129 | ∞ | 0.372 | 1.041 | -2.457 | 0.042 | ^*^ |
| Low / High | 0.671 | 0.149 | ∞ | 0.435 | 1.204 | -1.799 | 0.144 |  |
| Medium / High | 1.164 | 0.252 | ∞ | 0.762 | 2.060 | 0.703 | 0.482 |  |

| **Total bird richness** | | | | | | | |  |
| --- | --- | --- | --- | --- | --- | --- | --- | --- |
|  |  | | | | | | |  |
| **Contrast** | **Diff** | **SE** | ***df*** | **LCL** | **UCL** | **Z score** | **P value** |  |
| Control / Low | 0.1 | 0.21 | 27 | -0.50 | 0.70 | 0.475 | 1.000 |  |
| Control / Medium | -0.3 | 0.21 | 27 | -0.90 | 0.30 | -1.426 | 0.661 |  |
| Control / High | -0.3 | 0.21 | 27 | -0.90 | 0.30 | -1.426 | 0.661 |  |
| Low / Medium | -0.4 | 0.21 | 27 | -1.00 | 0.20 | -1.901 | 0.408 |  |
| Low / High | -0.4 | 0.21 | 27 | -1.00 | 0.20 | -1.901 | 0.408 |  |
| Medium / High | 0.0 | 0.21 | 27 | -0.60 | 0.60 | 0.000 | 1.000 |  |

| **Weekly bird richness** | | | | | | | | |
| --- | --- | --- | --- | --- | --- | --- | --- | --- |
|  |  | | | | | | |  |
| **Contrast** | **Ratio** | **SE** | ***df*** | **LCL** | **UCL** | **Z score** | **P value** |  |
| Control / Low | 0.727 | 0.115 | ∞ | 0.479 | 1.105 | -2.007 | 0.179 |  |
| Control / Medium | 0.623 | 0.095 | ∞ | 0.416 | 0.933 | -3.090 | 0.012 | ^*^ |
| Control / High | 0.664 | 0.103 | ∞ | 0.440 | 1.001 | -2.629 | 0.043 | ^*^ |
| Low / Medium | 0.856 | 0.121 | ∞ | 0.590 | 1.242 | -1.100 | 0.814 |  |
| Low / High | 0.913 | 0.131 | ∞ | 0.625 | 1.334 | -0.634 | 1.000 |  |
| Medium / High | 1.066 | 0.147 | ∞ | 0.741 | 1.534 | 0.464 | 1.000 |  |

| **Interactive effects of time and resources on bird richness** | | | | | | | | |
| --- | --- | --- | --- | --- | --- | --- | --- | --- |
|  |  | | | | | | |  |
| **Contrast** | **Diff** | **SE** | ***df*** | **LCL** | **UCL** | **Z score** | **P value** |  |
| Control / Low | -0.077 | 0.055 | ∞ | -0.218 | 0.064 | -1.405 | 0.801 |  |
| Control / Medium | -0.058 | 0.053 | ∞ | -0.194 | 0.079 | -1.085 | 1.000 |  |
| Control / High | -0.085 | 0.054 | ∞ | -0.223 | 0.054 | -1.576 | 0.691 |  |
| Low / Medium | 0.019 | 0.049 | ∞ | -0.105 | 0.144 | 0.400 | 1.000 |  |
| Low / High | -0.008 | 0.049 | ∞ | -0.135 | 0.119 | -0.158 | 1.000 |  |
| Medium / High | -0.027 | 0.048 | ∞ | -0.149 | 0.095 | -0.574 | 1.000 |  |

| **Weekly seed counts** | | | | | | | | |
| --- | --- | --- | --- | --- | --- | --- | --- | --- |
|  |  | | | | | | |  |
| **Contrast** | **Ratio** | **SE** | ***df*** | **LCL** | **UCL** | **Z score** | **P value** |  |
| Control / Low | 0.590 | 0.285 | ∞ | 0.165 | 2.110 | -1.091 | 1.000 |  |
| Control / Medium | 0.544 | 0.269 | ∞ | 0.148 | 2.000 | -1.231 | 1.000 |  |
| Control / High | 0.776 | 0.403 | ∞ | 0.198 | 3.050 | -0.488 | 1.000 |  |
| Low / Medium | 0.923 | 0.428 | ∞ | 0.272 | 3.130 | -0.174 | 1.000 |  |
| Low / High | 1.320 | 0.644 | ∞ | 0.362 | 4.790 | 0.561 | 1.000 |  |
| Medium / High | 1.439 | 0.698 | ∞ | 0.392 | 5.190 | 0.725 | 1.000 |  |

| **Interactive effects of time and resources on seed counts** | | | | | | | | |
| --- | --- | --- | --- | --- | --- | --- | --- | --- |
|  |  | | | | | | |  |
| **Contrast** | **Diff** | **SE** | ***df*** | **LCL** | **UCL** | **Z score** | **P value** |  |
| Control / Low | -0.043 | 0.021 | ∞ | -0.098 | 0.012 | -2.084 | 0.149 |  |
| Control / Medium | -0.049 | 0.020 | ∞ | -0.102 | 0.003 | -2.466 | 0.068 | ^†^ |
| Control / High | -0.064 | 0.021 | ∞ | -0.118 | -0.010 | -3.116 | 0.011 | ^*^ |
| Low / Medium | -0.006 | 0.017 | ∞ | -0.050 | 0.038 | -0.353 | 1.000 |  |
| Low / High | -0.021 | 0.017 | ∞ | -0.066 | 0.025 | -1.192 | 0.700 |  |
| Medium / High | -0.015 | 0.017 | ∞ | -0.058 | 0.029 | -0.885 | 0.752 |  |

| **Total seed richness** | | | | | | | |  |
| --- | --- | --- | --- | --- | --- | --- | --- | --- |
|  |  | | | | | | |  |
| **Contrast** | **Ratio** | **SE** | ***df*** | **LCL** | **UCL** | **Z score** | **P value** |  |
| Control / Low | 0.818 | 0.365 | ∞ | 0.252 | 2.650 | -0.450 | 1.0000 |  |
| Control / Medium | 0.500 | 0.203 | ∞ | 0.172 | 1.460 | -1.711 | 0.5227 |  |
| Control / High | 0.500 | 0.203 | ∞ | 0.172 | 1.460 | -1.711 | 0.5227 |  |
| Low / Medium | 0.611 | 0.232 | ∞ | 0.224 | 1.660 | -1.297 | 0.7791 |  |
| Low / High | 0.611 | 0.232 | ∞ | 0.224 | 1.660 | -1.297 | 0.7791 |  |
| Medium / High | 1.000 | 0.331 | ∞ | 0.418 | 2.390 | 0.000 | 1.0000 |  |

| **Per sample seed richness** | | | | | | | |  |
| --- | --- | --- | --- | --- | --- | --- | --- | --- |
|  |  | | | | | | |  |
| **Contrast** | **Ratio** | **SE** | ***df*** | **LCL** | **UCL** | **Z score** | **P value** |  |
| Control / Low | 0.771 | 0.358 | ∞ | 0.234 | 2.540 | -0.559 | 1.0000 |  |
| Control / Medium | 0.643 | 0.295 | ∞ | 0.198 | 2.090 | -0.965 | 1.0000 |  |
| Control / High | 0.687 | 0.322 | ∞ | 0.206 | 2.290 | -0.802 | 1.0000 |  |
| Low / Medium | 0.833 | 0.350 | ∞ | 0.284 | 2.450 | -0.435 | 1.0000 |  |
| Low / High | 0.891 | 0.384 | ∞ | 0.295 | 2.690 | -0.269 | 1.0000 |  |
| Medium / High | 1.069 | 0.454 | ∞ | 0.359 | 3.180 | 0.157 | 1.0000 |  |

| **Interactive effects of time and resources on per sample seed richness (Holm’s correction)** | | | | | | | | |
| --- | --- | --- | --- | --- | --- | --- | --- | --- |
|  |  | | | | | | |  |
| **Contrast** | **Diff** | **SE** | ***df*** | **LCL** | **UCL** | **Z score** | **P value** |  |
| Control / Low | -0.040 | 0.020 | ∞ | -0.092 | -0.011 | -2.053 | 0.160 |  |
| Control / Medium | -0.053 | 0.020 | ∞ | -0.103 | -0.003 | -2.784 | 0.027 | ^*^ |
| Control / High | -0.056 | 0.019 | ∞ | -0.106 | -0.006 | -2.950 | 0.019 | ^*^ |
| Low / Medium | -0.013 | 0.014 | ∞ | -0.048 | 0.024 | -0.913 | 0.744 |  |
| Low / High | -0.016 | 0.014 | ∞ | -0.052 | 0.020 | -1.156 | 0.744 |  |
| Medium / High | -0.004 | 0.013 | ∞ | -0.037 | 0.030 | 0.273 | 0.785 |  |
